# Comparative Transcriptome Analysis of mRNA and miRNA during the Development of Longissimus Dorsi Muscle of Gannan Yak and Tianzhu White Yak

**DOI:** 10.3390/ani14152278

**Published:** 2024-08-05

**Authors:** Yanmei Niu, Dashan Guo, Yali Wei, Jingsheng Li, Yanbin Bai, Zhanxin Liu, Xue Jia, Zongchang Chen, Liang Li, Bingang Shi, Xiaolan Zhang, Zhidong Zhao, Jiang Hu, Jiqing Wang, Xiu Liu, Shaobin Li

**Affiliations:** Gansu Key Laboratory of Herbivorous Animal Biotechnology, College of Animal Science and Technology, Gansu Agricultural University, Lanzhou 730070, China; niuym@st.gsau.edu.cn (Y.N.); guods@st.gsau.edu.cn (D.G.); weiyl@st.gsau.edu.cn (Y.W.); lijs@st.gsau.edu.cn (J.L.); baiyb@st.gsau.edu.cn (Y.B.); liuzx@st.gsau.edu.cn (Z.L.); jiax@st.gsau.edu.cn (X.J.); chenzongc@st.gsau.edu.cn (Z.C.); liliang@st.gsau.edu.cn (L.L.); shibg@gsau.edu.cn (B.S.); zhangxl@gsau.edu.cn (X.Z.); huj@gsau.edu.cn (J.H.); wangjq@gsau.edu.cn (J.W.); liux@gsau.edu.cn (X.L.); lisb@gsau.edu.cn (S.L.)

**Keywords:** Gannan yak, Tianzhu white yak, miRNA, RNA-seq, production performance

## Abstract

**Simple Summary:**

Skeletal muscle growth and development are closely related to the growth performance of livestock and poultry, and miRNAs are involved in skeletal muscle growth and development by negatively regulating target genes. In this study, the Gannan yak and the Tianzhu white yak which have large differences in production performance were used as research subjects. The difference of muscle fibers was analyzed by fiber staining. A total of 254 differentially expressed (DE) miRNAs were screened by transcriptome sequencing. Functional enrichment analysis revealed that the target genes were significantly enriched in muscle growth-related terms and pathways. By constructing a DE miRNA- DE mRNA interaction network, 18 key miRNAs related to muscle growth and development such as miR-3968-z, miR-2478-z, novel-m0143-3p, novel-m0024-3p, novel-m0128-5p, and novel-m0026-3p were screened. These results help us to explore the key influencing factors of skeletal muscle development in different breeds of yaks, and lay a foundation for further research on the molecular regulatory mechanisms of muscle growth and development.

**Abstract:**

The Gannan yak, a superior livestock breed found on the Tibetan Plateau, exhibits significantly enhanced body size, weight, and growth performance in comparison to the Tianzhu white yak. MiRNAs play a pivotal role in regulating muscle growth by negatively modulating target genes. In this study, we found the average diameter, area, and length of myofibers in Gannan yaks were significantly higher than those of Tianzhu white yaks. Further, we focused on analyzing the longissimus dorsi muscle from both Gannan yaks and Tianzhu white yaks through transcriptome sequencing to identify differentially expressed (DE)miRNAs that influence skeletal muscle development. A total of 254 DE miRNAs were identified, of which 126 miRNAs were up-regulated and 128 miRNAs were down-regulated. GO and KEGG enrichment analysis showed that the target genes of these DE miRNAs were significantly enriched in signaling pathways associated with muscle growth and development. By constructing a DE miRNA- DE mRNA interaction network, we screened 18 key miRNAs, and notably, four of the candidates (novel-m0143-3p, novel-m0024-3p, novel-m0128-5p, and novel-m0026-3p) targeted six genes associated with muscle growth and development *(DDIT4*, *ADAMTS1*, *CRY2*, *AKIRIN2*, *SIX1,* and *FOXO1*). These findings may provide theoretical references for further studies on the role of miRNAs in muscle growth and development in Gannan yaks.

## 1. Introduction

The Gannan yak and the Tianzhu white yak are both unique and high-quality resources living on the Tibetan Plateau. They play an important role in the economy and agriculture of the Tibetan Plateau by providing essential livestock products such as meat, milk, and wool [1]. The Tianzhu white yak, with a small population, is a local breed known for its wool and meat production [2], while the Gannan yak is an excellent local breed mainly for meat production [3]. According to the Third Census of Livestock and Poultry Genetic Resources in Gansu Province and the findings by Yang and Wang et al., adult Gannan yaks have a body height, body length, and body weight of 136.81 ± 4.58 cm, 149.93 ± 5.41 cm, and 314.79 kg, respectively. Its size, weight, and productivity are significantly better than that of the Tianzhu white yak [4,5]. Therefore, investigating the molecular regulatory mechanisms underlying muscle growth and development in these two yak breeds holds immense significance as it may provide valuable theoretical insights for genetic breeding and enhancement of yak populations.

The production performance of meat is a crucial economic indicator in beef cattle breeding and is intricately linked to the growth and development of skeletal muscle tissue [6]. Skeletal muscle growth is primarily categorized into two distinct stages: prenatal and postnatal [7]. During the prenatal period, skeletal muscle growth is determined by myofibers’ quantity, while during the postnatal period, it is influenced by myofibers’ diameter and volume [8,9]. Myofibrils serve as fundamental components of muscles, directly impacting meat quality and production [10]. Furthermore, numerous studies have demonstrated that multiple signal transduction pathways and transcription factors play pivotal roles in regulating skeletal muscle growth and development. For instance, the AMPK signaling pathway plays a critical role in the reparative process following muscle injury [11,12]. *PAX3* and *PAX7* synergistically collaborate with myogenesis regulators such as *MYF5*, *MYOD*, *MYOG*, and *MRF4* to regulate intricate processes such as skeletal muscle proliferation and differentiation [13]. Additionally, specific mRNAs in skeletal muscle tissue including *SIX1* [14], *E2F3* [15], *IGFs* [16], and *MEF2* [17] influence skeletal muscle growth. Moreover, non-coding RNAs (ncRNAs), particularly microRNAs (miRNAs), significantly impact skeletal muscle growth through various biological processes regulated via multiple mechanisms.

MiRNAs’ highly conserved endogenous non-coding RNAs intricately regulate numerous protein-coding genes and play a pivotal role in gene regulation. Previous studies have revealed that miRNAs exert intricate regulatory effects on a diverse array of biological processes, encompassing muscle growth, development, proliferation, and differentiation [18,19]. For instance, the researchers identified a specific binding mechanism of miR-133, miR-1, miR-206, and miR-151-3p to target genes that influences muscle growth and development [20]. Deng et al. reported an up-regulation of muscle-specific miR-1 and miR-133 expression during skeletal muscle differentiation [21]. Research has found high expression of miR-206 is highly expressed in slow muscle [22], which was shown to modulate endogenous *HDAC4* levels in mouse muscles through overexpression or inhibition resulting in decreased or increased levels, respectively [23]. Overexpression of miR-151-3p not only resulted in an increase in myoblast proliferation but also led to the down-regulation of slow muscle genes in both C2C12 myotubes and primary cultures [24]. Furthermore, it has been discovered that miR-199b [25] and miR-34c [26] suppress the proliferation and differentiation of porcine skeletal muscle stem cells by targeting *JAG1* and *NOTCH1*, respectively. Meanwhile, miR-128, miR-143, and miR-377 hinder the expansion and specialization of bovine skeletal muscle stem cells (SMSCs) by targeting *SP1*, *IGFBP5,* and *FHL2,* respectively [27,28,29]. It is intriguing that, Guo et al. conducted transcriptome sequencing on the longissimus dorsi muscle tissues from Gannan yak and Jeryak, leading to the identification of 14 key miRNAs, including miR-2478-z and novel-m0036-3p potentially involved in muscle growth and development [11]. However, limited attention has been devoted to investigating individual growth and development differences between Gannan yaks and Tianzhu white yaks. Furthermore, there are still significant knowledge gaps regarding the role of miRNA in muscle growth and development in both breeds of yaks that require further exploration.

Therefore, in this study, we utilized four-year-old adult Gannan yaks and Tianzhu white yaks to investigate the disparities in myofiber tissue characteristics between these two breeds by Hematoxylin and Eosin staining, as well as fast and slow muscle fluorescence staining techniques. Additionally, we assessed the expression patterns of miRNAs in the longissimus dorsi muscle of both breeds using transcriptome sequencing, subsequently constructing networks depicting interactions between differentially expressed miRNA and mRNA. These findings not only facilitate our exploration of pivotal factors influencing skeletal muscle development across diverse yak breeds but also establish a robust foundation for further research on the molecular regulatory mechanisms governing muscle growth and development.

## 2. Materials and Methods

### 2.1. Laboratory Animals and Sample Collection

Three Gannan yaks (male yak, four-year-old adult) were obtained from Cooperation City Gannan Tibetan Autonomous Prefecture, Gansu Province. We identified it as Group M, and three Tianzhu white yaks (male yak, four-year-old adult) were obtained from Wuwei City, Gansu Province. We identified it as Group A. They were provided with ad libitum access to feed and water under the same feeding conditions. Following approval from the Experimental Animal Ethics Committee of Gansu Agricultural University, adult male yaks with similar body weights were selected and subjected to fasting treatment before being slaughtered in the abattoir. The slaughter was done by electroshock and bloodletting through the carotid artery. Samples of longissimus dorsi muscle were collected immediately after slaughter, temporarily stored in liquid nitrogen, and brought back to the laboratory for storage at −80 °C.

### 2.2. Analysis of Muscle Fiber Characteristics

To observe the morphological differences in muscle histology, techniques such as HE staining and fast/slow muscle fluorescence staining were utilized. The collected tissue of the longissimus dorsi muscle was initially fixed in a 4% neutral paraformaldehyde solution, and then stained by HE staining according to Shi’s method [30]. The fixed muscle tissue was removed to make paraffin sections with thickness of 5 µm. The dyeing process involved sequential steps including dehydration, embedding, slicing, dyeing, sealing, and image acquisition. Subsequently, the tissue sections were immersed in a container with an EDTA antigen recovery buffer for preparation. After cooling, the slide was soaked in PBS (pH = 7.4) and cleaned by stirring with a dyeing shaker. The blocking serum, primary antibody, secondary antibody, and DAPI staining solution were added successively. The cells were incubated at room temperature under light for 10 min and then washed with PBS. Self-fluorescence quencher was added before sealing. The sections were observed under a fluorescence microscope, and subsequent images were captured. The results indicate that DAPI-stained nuclei exhibited blue fluorescence, while fast muscle fibers showed red fluorescence, and slow muscle fibers showed green fluorescence.

Specific areas were selected for data analysis using the Use Case Viewer 2.2 scanner, with eight areas per slice. The images were then imported into Image-Pro Plus 6.0 software for detailed analysis to find out their diameter, area, and type. Finally, IBM SPSS Statistics 26 software was used to conduct an independent sample *t*-test on the measurement results, and the data were expressed as mean ± standard error.

### 2.3. Total RNA Extraction and Sequencing

To isolate total RNA from longissimus dorsi muscle tissue samples, a Trizol kit (Invitrogen, Carlsbad, CA, USA) was utilized. The tissue was initially ground to the size of a grain of rice in liquid nitrogen and then transferred to a centrifuging tube where 1000 µL Trizol was added and thoroughly mixed. After standing for 5 min, the tissue was centrifuged at 12,000× *g* for 5 min, and the supernatant was gently aspirated for follow-up experiments. Subsequently, a fifth of the chloroform was added to the lysate and thoroughly mixed. This mixture was centrifuged for 15 min. Then the supernatant was aspirated again, and 500 µL of isopropyl alcohol was added. This mixture stood for 10 min before being centrifuged at 12,000× *g* for another 10 min. The supernatant was extracted and added to 1000 µL (80% concentration) of pre-cooled ethanol solution. The extraction RNA was washed and centrifuged at 7500× *g* for 5 min resulting in precipitation. It was then dried at room temperature before adding enzyme-free water to form a final volume of 20 µL. After extraction, the RNA concentration and purity were determined using Nanodrop 2000 (Thermo Scientific, Waltham, MA, USA). Subsequently, small RNA fragments were isolated using polyacrylamide gel electrophoresis (PAGE). The purified sequences were amplified by PCR followed by construction of a cDNA library. After quality assessment had been conducted on this, library sequencing took place on an Illumina NovaSeq6000 instrument (Guangzhou, China).

### 2.4. Data Quality Control and Differential miRNA Screening and Identification

To obtain clean labels, we processed the raw sequencing reads as follows: First, low-quality readings containing more than one base or unknown nucleotide (N) with Q value ≤ 20 were eliminated; Secondly, readings missing a 3′ adapter and containing a 5′ adapter were discarded, and readings containing both 3′ and 5′ adapters but were missing small RNA fragments in between were filtered out; finally, readings containing polyA in small RNA fragments were ignored and readings shorter than 18 nt (excluding aptamers) were discarded. To identify and exclude non-coding RNAs, we utilized GenBank (v209.0) and Rfam (v11.0). Additionally, we employed (LU_Bosgru_v3.0) for yak reference genome comparison. New miRNAs were predicted using mirdeep2 (v2.0.0.7) software [31] and miRNA expression levels were calculated using TPM. Heat maps of different types of miRNAs were generated, and miRNAs with similar expression patterns were clustered and analyzed. Differential expression analysis was conducted using DESeq2 (V1.20.0) software [32]. We considered miRNAs to be differentially expressed when |log2FC| > 1 and *p* < 0.05.

### 2.5. (DE) miRNA Target Gene Prediction and Functional Enrichment Analysis

We utilized Miranda (v3.3a) [33] and TargetScan (v7.0) [34] software to predict the target genes of differentially expressed miRNAs. The overlapping results from both datasets are considered as the final miRNA target genes. Subsequently, GO enrichment analysis was conducted on the database (http://www.geneontology.org/, accessed on 13 July 2023) using the hypergeometric test. KEGG enrichment analysis was also performed on the database (http://www.kegg.jp/kegg/, accessed on 13 July 2023) using the hypergeometric test. Finally, GO entries of differentially expressed miRNA target genes and KEGG pathways were deemed significantly enriched when the *p* < 0.05.

### 2.6. (DE)miRNA-(DE)mRNA Regulatory Network Analysis

We performed a comprehensive analysis using RNA-seq (accession number: PRJNA1108616, PRJNA1023680, and PRJNA1023693) and intersected predicted miRNA target genes with mRNA sequencing results. Subsequently, DEMs related to muscle growth and development were determined by reference to relevant literature. The miRNA-mRNA interaction network was constructed using Cytoscape software (v3.6.0).

### 2.7. Validation of qRT-PCR for (DE) miRNAs

We randomly selected seven (DE)miRNAs to verify the reliability of the sequencing data. Forward primers for miRNAs using miRNA Design V1.01 are shown in (Table 1). Total RNA was then extracted from the samples using Trizol reagent (Invitrogen, Carlsbad, CA, USA) with U6 being used as the internal reference gene. The miRNA first-strand cDNA synthesis kit (Transgen Biology, Beijing, China) was used for reverse transcription and real-time fluorescence quantitative PCR.

Additionally, to verify whether these (DE) miRNAs are involved in muscle growth and development of Gannan yak, we analyzed miRNA expression levels in the liver, heart, kidney, lungs, spleen, subcutaneous fat, and longissimus dorsi muscle.

All qRT-PCR reactions were conducted using an ABI Prism 7500 Real-Time PCR System (Applied Biosystems, Foster City, CA, USA), with four biological replicates and *U6* as an endogenous gene. The 2^−ΔΔCT^ method was employed for data analysis. Graphs for all experiments were generated using GraphPad Prism 9 (GraphPad Software, San Diego City, CA, USA).

## 3. Results

### 3.1. Analysis of Dyeing Characteristics of Muscle Fibers

**Figure 1:**
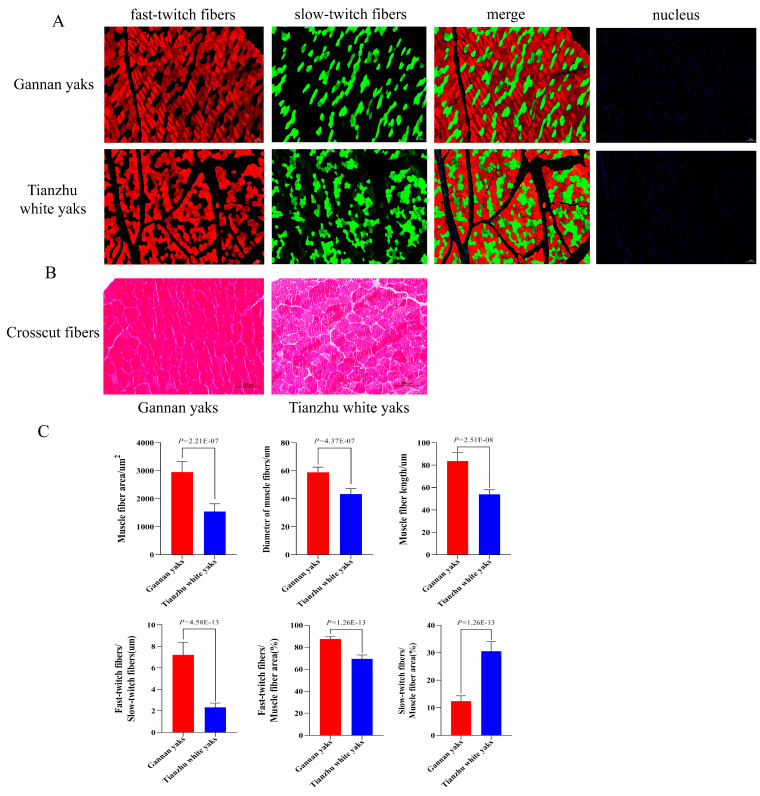
Analysis of muscle fibers staining results. (**A**) Fluorescent staining of fast and slow muscle fibers (red fluorescence for fast muscle; green fluorescence for slow muscle; blue fluorescence for nuclei). (**B**) HE staining of paraffin sections (red represents individual muscle fibers). (**C**) Comparisons of muscle fibers characteristics of different breeds of bovine (blue represents Gannan yak; red represents Tianzhu white yak).

To analyze the differences in muscle fiber, myofibers from Gannan yaks and Tianzhu white yaks were subjected to fast and slow fluorescence staining (Figure 1A), as well as HE staining (Figure 1B). The stained samples were then analyzed using IPP (Image-Pro Plus 6.0) software. The results reveal that the average diameter, area, and length of myofibers in Gannan yaks are significantly higher than those of Tianzhu white yaks (*p* < 0.01). Furthermore, the ratios between fast muscle and slow muscle, the fluorescence intensity of fast muscle and slow muscle, and fluorescently stained fast muscle to myofibers’ area were all significantly greater in Gannan yaks compared to Tianzhu white yak (*p* < 0.01). Conversely, the ratio between fluorescently stained slow muscle and myofibers’ area was smaller in Gannan yaks than that of Tianzhu white yak (*p* < 0.01) (Figure 1C).

### 3.2. Identification and Characterization of miRNAs

**Figure 2:**
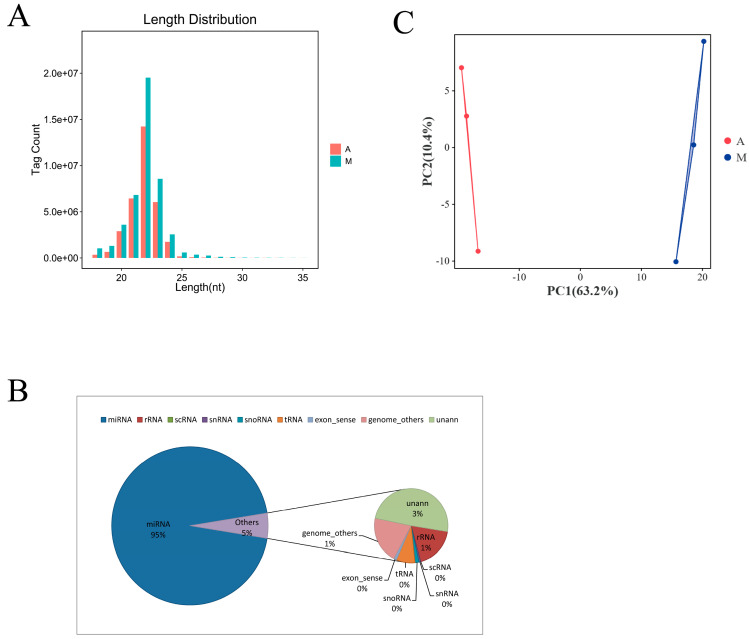
Small RNA sequencing results. (**A**) Small RNA length distribution; (**B**) Small RNA type distribution; (**C**) Principal component analysis (PCA) results.

To investigate the (DE) miRNA during muscle development in Gannan yak and Tianzhu white yak, RNA-seq technology was used to sequence six longissimus dorsi muscle tissues from Gannan yaks (M) and Tianzhu white yaks (A). A total of 11,845,237, 11,258,234, 9,755,904, 17,408,914, 14,015,050, and 16,597, 591 clean readings were produced in group M and group A, respectively. After quality control, a total of 11,432,116, 11,881,484, 9,361,019, 16,255,077, 13,362,108, and 15,172,291 clean tags were acquired and utilized for subsequent examination (Table 2). Character analysis revealed that the small RNA lengths were mainly distributed between 18-24 nt with a length of 22 nt accounting for the largest proportion between the two species (Figure 2A). Subsequently, all the clean tags were compared with GenBank and Rfam, and it was found that after filtering out some low-quality sequences and splice sequences, 95% of the clean reads were considered to be miRNAs, and the rest were rRNAs, scRNAs, snRNAs, snoRNAs, tRNAs, and unanns. (Figure 2B, Table S1), Of these, 80.23% and 72.17% of clean tags were mapped to the yak reference genome in A and M, respectively (LU_Bosgru_v3.0) (Table S2). To further validate the quality of the sequencing data, the differences between the two samples and the similarities within the same species were revealed using principal component analysis plots (Figure 2C), and from these results, it was found that the sequencing data were of high quality.

### 3.3. Differential Expression Analysis of miRNAs

**Figure 3:**
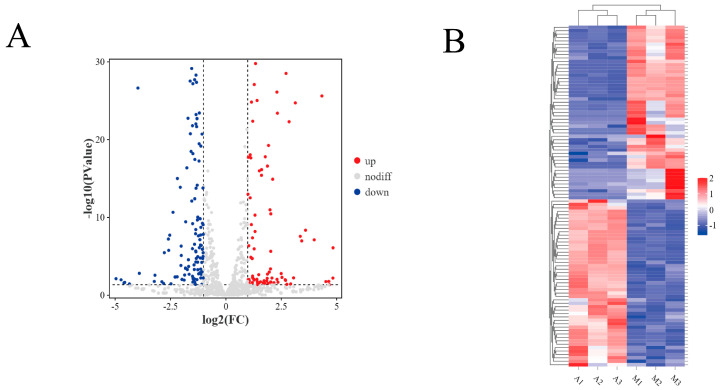
Analysis of differentially expressed miRNA. (**A**) Volcano plots illustrate the differential expression of miRNAs. In Figure (**A**), the blue dot on the left indicates down-regulated miRNAs, the gray dot in the middle indicates non-significant differences, and the red dot on the right indicates up-regulated miRNAs. (**B**) Cluster plots display differential expression miRNA, with red indicating high expression and blue indicating low expression.

We utilized DESeq2 (V1.20.0) software to analyze the miRNAs in group M and group A and identified 254 differentially expressed miRNAs, comprising 204 known miRNAs and 51 newly discovered ones. In comparison to Tianzhu white yaks, Gannan yaks exhibited a significant down-regulation of 128 (DE)miRNAs and an up-regulation of 126 (DE)miRNAs in the longissimus dorsi muscle (Figure 3A). The clustering results of different miRNAs were clustered in the two branches of A and M, respectively (Figure 3B).

### 3.4. Differential Expression miRNA GO and KEGG Analysis and Target Gene Prediction

**Figure 4:**
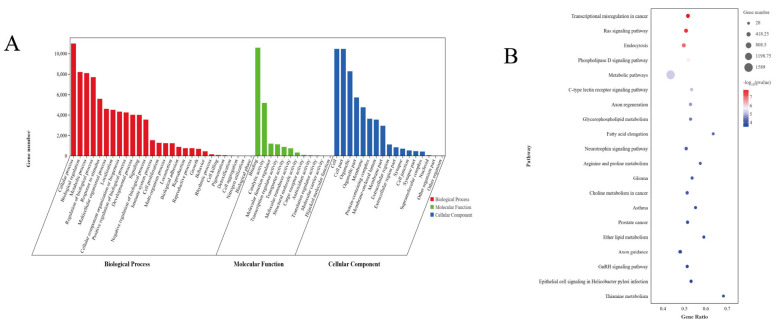
GO and KEGG analysis of differentially expressed miRNA. (**A**) GO enrichment analysis of differential miRNAs, (**B**) Top 20 KEGG-enriched pathways for differential miRNAs.

To further investigate the potential mechanisms of (DE)miRNAs in skeletal muscle development, we utilized Targetscan and miRanda software to predict their target genes. The analysis revealed that 254 (DE)miRNAs were associated with 17,365 predicted target genes (Table S3). GO enrichment analysis indicated significant enrichment (*p* < 0.05, Table S4) of these target genes in 3177 functional categories. Among these, 266 GO terms were enriched in the cellular component, including terms such as cell, actin filament bundle, growth factor complex, Wnt signaling body, and PRCI complex; and 569 GO terms enriched in molecular functions with transcriptional co-regulatory activity, growth factor binding, catalytic activity, transcription factor binding, co-receptor activity involved in the Wnt signaling pathway, and biological process. The classification was expanded with 2342 GO terms, encompassing animal organ development, cell proliferation, skeletal muscle tissue development, skeletal muscle fibers development, and regulation of cell growth (Figure 4A). KEGG analysis revealed that the target genes of (DE)miRNAs were significantly enriched in 115 signaling pathways (*p* < 0.05, Table S5), primarily involved in the MAPK signaling pathway, Wnt signaling pathway, Ras signaling pathway, and fatty acid metabolism (Figure 4B).

### 3.5. Constructing miRNA-mRNA Interaction Networks

**Figure 5:**
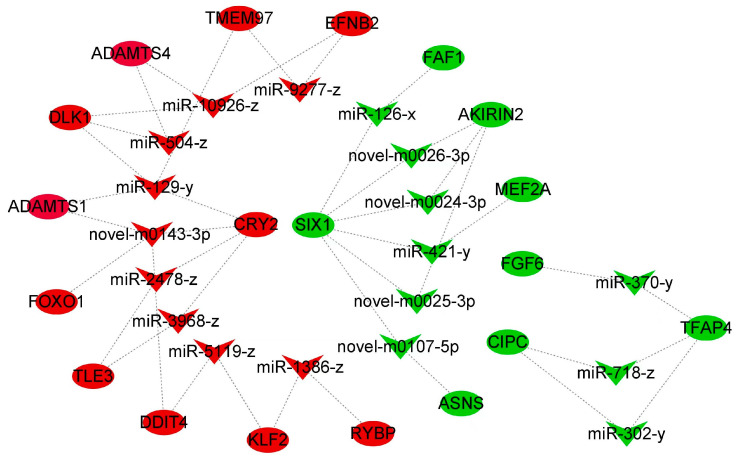
Interaction network of Gannan yak and Tianzhu white yak (DE)miRNA-DE mRNA. In the network, red circles represent down-regulated mRNAs and red arrows represent up-regulated miRNA; green circles represent up-regulated mRNAs and green arrows represent down-regulated miRNA.

Based on the negative regulation of miRNAs on target genes, we conducted a comprehensive analysis of 254 DE miRNAs and RNA-seq screened DEMs to identify key miRNAs. In order to investigate the mechanism underlying muscle growth in Gannan yak, we further explored the relationship between 8377 pairs of miRNA-mRNA interactions (Table S6). Subsequently, by reviewing literature and investigating target gene prediction results, we identified 17 DEMs and 18 key DE miRNAs associated with muscle growth. Based on these findings, an interaction network was constructed between the DE miRNAs and DE mRNAs (Figure 5), demonstrating their ability to target genes related to muscle growth. For example, significantly up-regulated miR-129-y targets *DLK1*, *ADAMTS1*, *CRY2*, *TMEM97*, and other genes involved in muscle growth. Additionally, significantly down-regulated novel-m0126-5p targets *SIX1* and *FGF6* which are also related to muscle growth. These findings suggest that these DE miRNAs may play a role in regulating muscle growth by modulating the expression of their target genes.

### 3.6. qRT-PCR Validation of Differentially Expressed miRNAs

**Figure 6:**
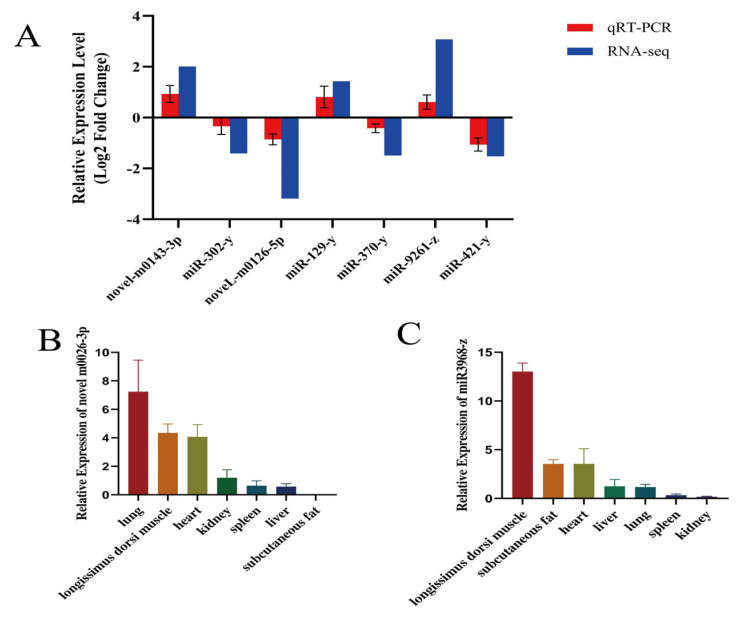
DE miRNA qRT-PCR validation results. (**A**) miRNA expression in groups A and M. (**B**) Tissue expression profile of novel-m0026-3p. (**C**) Tissue expression profile of miR-3968-z. Tissue expression profiles used were (subcutaneous fat, longissimus dorsi muscle, heart, kidney, liver, spleen, and lung). Data represent mean ± standard error.

To assess the reliability of the sequencing data, we randomly selected seven differentially expressed miRNAs for qRT-PCR analysis: miR-129-y, novel-m0143-3p, miR-9261-z, miR-421-y, novel-m0126-5p, miR-370-y, and miR-302-y (Figure 6A). The results demonstrate that the qRT-PCR findings are consistent with the sequencing data. To further validate the potential involvement of these miRNAs in muscle growth and development, we chose to investigate the newly discovered novel-m0026-3p (Figure 6B) and miR-3968-z which exhibited the highest differential expression (Figure 6C). We utilized qRT-PCR to confirm their expression levels in various tissues.

## 4. Discussion

The Gannan yak exhibits superior body size, weight, and productivity compared to Tianzhu white yak. In order to investigate the molecular mechanism underlying this high productivity, we characterized myofibers and performed transcriptome sequencing to examine the role of miRNA in muscle growth and development. Previous studies have emphasized the significance of myofibers in meat quality and yield by directly influencing muscle composition [10]. Smaller diameter and area of myofiber are associated with increased tenderness in meat [35]. Our study revealed that Gannan yaks exhibited significantly larger average myofibers’ diameter, area, and length compared to Tianzhu white yaks, indicating greater hypertrophy and reduced tenderness in their myofibers. Additionally, the ratios between fast and slow muscle fibers also impact meat production performance [36]. The ratios between fluorescently stained fast muscle fibers and total muscle fibers area were higher in Gannan yaks than in Tianzhu white yaks. Conversely, the ratios between fluorescently stained slow muscle fibers and total muscle fiber area were lower in Gannan yaks compared to Tianzhu white yaks. These differences may account for variations observed in meat production performance between Gannan yak and Tianzhu white yak breeds while providing a foundation for studying their molecular regulatory mechanisms.

MiRNAs play key roles in various aspects of muscle biology, including the regulation of muscle growth, development, proliferation, and differentiation [37]. In this study, a total of 254 differentially expressed miRNAs were identified, including 204 known miRNAs and 51 newly discovered miRNAs. Functional enrichment analysis revealed that these differentially expressed miRNAs primarily involved in GO entries are associated with Wnt signaling pathway co-receptor activity, cell proliferation, skeletal muscle tissue development, skeletal muscle fiber development, as well as Wnt semaphore. Moreover, KEGG enrichment pathway analysis revealed that the target genes of differentially expressed miRNAs were significantly enriched in the MAPK signaling pathway, Wnt signaling pathway, Ras signaling pathway, and fatty acid metabolism pathway. It has been established that the MAPK signaling pathway regulates diverse biological processes of myoblasts, such as proliferation, growth, migration, differentiation, apoptosis, and more specifically, muscle cell hypertrophy [38]. The Wnt signaling pathway plays a crucial role not only in skeletal muscle regeneration but also in the regulation of muscle fiber typing through transactivation [39]. Activation of the Wnt signaling promotes muscle fibrogenesis, particularly through the Wnt/β-catenin pathway, resulting in alterations in the expression of specific extracellular matrix components [40]. Conversely, the Ras signaling pathway exerts potent inhibitory effects on skeletal myogenesis [41], thereby impending skeletal muscle growth and development. Furthermore, miR-2478-z has been found to exhibit higher expression levels in muscle tissues based on analyses conducted across various tissues, including heart, liver, and longissimus dorsi muscle [11]. Further analysis is required to elucidate the functions of newly discovered miRNAs such as novel-m0026-3p and novel-m0143-3p.

In order to further explore candidate miRNAs associated with muscle growth in Gannan yak, we performed integrated analysis and constructed DE miRNA -DE mRNA interaction networks. It is noteworthy that, the expression of certain newly identified miRNAs exhibited differences between the two yaks. Therefore, we hypothesize that these novel miRNAs may modulate muscle growth and development by regulating their target genes. Specifically, novel-m0024-3p and novel-m0026-3p were found to co-target *SIX1* and *AKIRIN2*, while novel-m0128-5p and novel-m0143-3p were observed to co-target *ADAMTS1* and *CRY2* within the interaction network. Previous studies have demonstrated that *SIX1* functions as a transcription factor crucial for skeletal muscle development [14], Additionally, *SIX1* collaborates with cofactors (Eya1, Eya3, and Dach2) to regulate skeletal muscle fibers type and development. Further investigations on flounder have revealed that *SIX1* is expressed in both fast-twitch and slow-twitch muscle fibers, but exhibits higher accumulation in the nuclei of fast-twitch fibers [42]. Moreover, the overexpression of *SIX1* in the skeletal system of mice not only enhanced the fusion of adult myoblast but also increased the number of nuclei per myotube [43]. In porcine skeletal muscle, *AKIRIN2* has been identified as an important factor in muscle development and composition, promoting slow expression of myosin heavy chain in satellite cells through CAN/NFATC1 signaling [44,45,46]. Additionally, overexpression of *ADAMTS1* in macrophages in vivo enhances satellite cell activation and promotes muscle regeneration [47]. This phenomenon was further confirmed in mouse skeletal muscle, where *ADAMTS1* overexpression promoted skeletal myocyte activation and muscle regeneration [48]. *CRY2* acts as an inhibitor of circadian rhythm, and its absence promotes the regeneration of myogenesis by enhancing *PAX7* expression and satellite cell proliferation. Furthermore, it has been demonstrated that *CRY2* directly targets miR-7-5p to induce osteoblast differentiation, leading to up-regulation of Runx2, ALP, collagen type I alpha 1 (Col1a1), and OCN expression. This highlights the targeting relationship between miRNAs and differential genes, ultimately promoting skeletal muscle growth and development [49,50].

Furthermore, miRNA regulates gene expression by binding to the 3′ untranslated region (3′UTR) of target mRNA, leading to mRNA cleavage or translation inhibition [20]. This phenomenon was also reflected in our reciprocal network, where we identified *SIX1* and *AKIRIN2* as up-regulated genes, and *DAMTS1* and *CRY2* as down-regulated genes. Therefore, it is speculated that the newly identified miRNAs may influence the growth and development of Gannan yak muscle by promoting or suppressing the expression of these differential genes. However, this study was employed a limited number of biological replicates for each group. In accordance with principle of repetition strength, it was observed that a higher repetition strength resulted in increased measurements, whereas a lower repetition strength leads to fewer measurements. This phenomenon is also evident in our principle of replication component analysis (PCA)diagram, which exhibits substantial difference between the two breeds and minimal variation within the same breed. We anticipate that these findings can serve as a foundation for further investigation into crucial miRNAs involved in the skeletal muscle development of Gannan yak. Additionally, this study primarily focused on the identification of key miRNAs and lacked functional validation. Future studies, should be aimed to verify, the targeting relationship between miRNAs and mRNAs as well as investigate. The potential regulatory mechanisms underlying these distinct miRNAs, thereby elucidating their specific roles in muscle growth and development.

## 5. Conclusions

In conclusion, we observed a significant increase in both muscle fiber area and length in Gannan yaks, indicating their enhanced muscle growth and development. Furthermore, we utilized the identified key miRNAs and their corresponding target genes to establish an interaction network associated with yak muscle growth and development. These discoveries significantly advanced our understanding of the genetic mechanisms underlying muscular traits in yaks and provided valuable insights as well as potential targets for genetic improvement programs in yak breeding.

## Figures and Tables

**Table 1:** Primer Sequence table. miRNA primer information for qRT-PCR.

miRNAs	Forward (5′ → 3′)	Reverse (5′ → 3′)
miR-129-y	AAGCCCTTACCCCAAAAAGGTAT	
novel-m0143-3p	CCACCAGGCCTGCAGCTCCGCC	
miR-9261-z	GCGGTGGGGCGCGGGACA	
miR-421-y	ATCAACAGACATTAATTGGGCGC	
novel-m0126-5p	CACTGAGACTCGCAGAAGCG	
miR-370-y	GCCTGCTGGGGTGGAACCTGGT	
miR-302-y	TAAGTGCTTCCATGTTTTAGTAG	
*U6*	GGAACGATACAGAGAAGATTAGC	TGGAACGCTTCACGAATTTGCG

**Table 2:** Data quality control table. Results of raw reads after quality control.

ID	Clean Reads	High Quality	3′Adapter Null	Insert Null	5′Adapter Contaminants	PolyA	Clean Tags
A1	11,845,237	11,752,376	5365 (0.0457%)	46,810 (0.3983%)	8035 (0.0684%)	71 (0.0006%)	11,432,116
	(100%)	(99.2160%)					(96.5123%)
A2	11,258,234	12,140,269	8840 (0.0728%)	34,315 (0.2827%)	4759 (0.0392%)	69 (0.0006%)	11,881,484
	(100%)	(99.0377%)					(96.9266%)
A3	9,755,904	9,634,408	3962 (0.0411%)	32,486 (0.3372%)	4045 (0.0420%)	52 (0.0005%)	9,361,019
	(100%)	(98.7546%)					(95.9523%)
M1	17,408,914	17,260,011	9612 (0.0557%)	108,519 (0.6287%)	31,981 (0.1853%)	321 (0.0019%)	16,255,077
	(100%)	(99.1447%)					(93.3721%)
M2	14,015,050	13,899,769	6900 (0.0496%)	54,327 (0.3908%)	13,958 (0.1004%)	162 (0.0012%)	13,362,108
	(100%)	(99.1774%)					(95.3411%)
M3	16,597,591	16,448,657	10,968 (0.0667%)	83,221 (0.5059%)	20,095 (0.1222%)	247 (0.0015%)	15,172,291
	(100%)	(99.1027%)					(91.4126%)

## Data Availability

The RNA-seq data from this study is available in the GenBank Sequence Read Archive (SRA) database with accession numbers PRJNA1051387, PRJNA1108616, PRJNA1023680, and PRJNA1023693.

## Supplementary Materials

The following supporting information can be downloaded at https://www.mdpi.com/article/10.3390/ani14152278/s1: Table S1: Distribution of small RNA types in Gannan yaks and Tianzhu white yaks; Table S2: Reference genome comparison rate; Table S3: Prediction of target genes of differentially expressed miRNAs; Table S4: GO enrichment analysis of differentially expressed miRNA target genes; Table S5: KEGG enrichment analysis of differentially expressed miRNA target genes; Table S6: Negative targeting relationship pairs of differentially expressed mRNAs and differentially expressed miRNAs.
